# The Influences of gradient color on the weight perception and stability perception: A preliminary study

**DOI:** 10.1177/20416695231197797

**Published:** 2023-08-30

**Authors:** Xing Xu, Jieying Zhang, Qi Zhu, Tiansheng Xia

**Affiliations:** Guangdong International Center of Advanced Design, 47870 Guangdong University of Technology, Guangzhou, China

**Keywords:** gradient color, stability perception, weight perception, sensory marketing

## Abstract

Gradient colors are widely used in product design. The variation of gradient colors muting a color as a series of steps from bright to dull creates a soft and gradual impression while also affecting people's perceptions. This study manipulates the types of gradient colors to explore the relationship between color gradients and perception of stability to determine whether weight perception plays a role. In the case of controlling for aesthetic differences, the study manipulated two types of color gradients (dark colors fading upward from the bottom versus downward from the top) and measured the perceptions of product stability. In the same hue, an upward gradient gives a stronger perception of stability. In addition, gradient colors significantly influence women's perception of stability more than men's. The study also investigated the mediating effect of weight perception: participants evaluated color fading-upward products with less weight relative to fading-downward colors. Furthermore, dark colors fading upward from the bottom lead to a stronger perception of weight, increasing the stability perception of the object. Finally, to aid future research, we discuss the practical implications of the current findings for areas such as sensory marketing, as well as possible directions for future research.

Today, more and more products use gradient colors to attract consumers, but rarely research has been done on the perceptual tendencies they bring to people. Specifically, the product's appearance can evoke people's perception of its physical features (Toufani et al., 2017). Stability is one of the important attributes of the product, consumers tend to take the stability of the product as an important factor in their decision when purchasing products (Wang, 2020). Interestingly, in Bullough's experiment, compared to 5 ft of red painted underneath 5 ft of pink, the effect of 5 ft of red painted on the wall above 5 ft of pink would give the majority of people an uncomfortable feeling of instability or lack of balance (top heaviness) (Bullough, 1907). Since the combination of pink and red colors can be regarded as a vertical arrangement of red brightness variation. Münsterberg observed that compared with the horizontal arrangement of elements, the perceptual stability of vertical arrangement is stronger (Münsterberg et al., 1894). This provides us with a direction to explore the relationship between color brightness gradient and stability perception.

Gradient color can usually be divided into hue gradient and brightness gradient. One of the forms of brightness gradient is in the same hue changed by mixing it with white (to form a tint) or with black (to form a shade) in varying proportions (Elliot & Maier, 2014; Stone et al., 2006). The assumption that the brightness of color has apparent weight is based on early studies, which generally found that “dark” colors appear heavier than “light” colors (Bullough, 1907; DeCamp, 1917; Löffler, 2017; Monroe, 1925; Payne, 1958, 1961; Warden & Flynn, 1926). In other words, colors with low relative brightness/low brightness appear heavier, while colors with high relative brightness/high brightness appear lighter (Löffler, 2017). This is due to the fact that the main factors affecting the heavy-light in visual perception include the brightness of the color. The gradation of color brightness seems to induce the optical illusion of uneven weight distribution, which in turn affects the perception of object stability. In color gradient relationships, color shade transitions often have the metaphor of a density/weight transition, and this connection may stem from the link between material density and surface color in nature. In nature, denser and heavier objects tend to appear darker (Alexander & Shansky, 1976; Bullough, 1907). Just as the actual density of objects determines their physical weight (Amazeen & Turvey, 1996; Saccone et al., 2019), humans might maintain a visual perceptual processing mechanism for assessing the stability of objects through the representation of relative material density (Lupo, 2015). Thus, the gradient form of brightness can also be viewed as a transition from high color density to low color density, which would create the visual-weight illusion and forms a metaphor for stability.

Moreover, the omnipresence of a brightness gradient approximately running from dark (below) to light (top) in the arts suggests that it may be a template in the psychogenesis of people's general visual awareness (Koenderink et al., 2015). And Koenderink et al. (2015) confirmed in the results of the psychological occurrence of people's visual awareness that the black-white gradient correlates well with the low (below)-high (top) position in the image. The environment we live in is dominated by gravity, and the placement of the heavy on the bottom and the light on top is an instinct of natural experience, as well as our bodily perception. If we feel “top-heavy,” it is often accompanied by an unstable state of the body. This is because the information of the body is contained in the representations that constitute cognition (Foglia & Wilson, 2013). According to the above assumptions, Bullough's experimental results can also be explained by the visual illusion of uneven weight spread caused by the way the color shades are arranged, thus creating the impression of instability. Since 5 ft of red is painted on the wall above 5 ft of pink makes the top of the graphic weight distribution appear heavy while the bottom appears light, an impression of instability is created for the whole graphic.

Gradient color, an increasingly widely used form of product appearance expression, surprisingly, little theoretical or empirical work has been conducted to date on the influence of gradient color on stability perception. And there seems to be a strong correlation between color gradient type and stability perception, as well as weight perception and stability perception. Therefore, this study selects the monochrome brightness gradient type, i.e., dark color from the bottom gradient to light color on the top and light color from the bottom gradient to dark color on the top (as in Figure 1) as the independent variable and people's perception of product stability as the dependent variable, in which product weight perception is selected as the mediating variable. We hypothesize that the monochrome brightness gradient type of transition from a bottom dark color to top light color brings stronger weight perception and thus increases people's perception of object stability, and the research framework is shown in Figure 2, with the following hypothesis:

H1: Dark upward gradient of monochrome brightness gradient type (dark color transitioning from the bottom to the top light color) brings a stronger perception of stability.
H2: Dark upward gradient of monochrome brightness gradient type (dark color transitioning from the bottom to the top light color) brings a stronger perception of weight.
H3: The weight perception caused by the monochrome brightness gradient type plays a mediating role in the process of stability perception.

**Figure 1.**
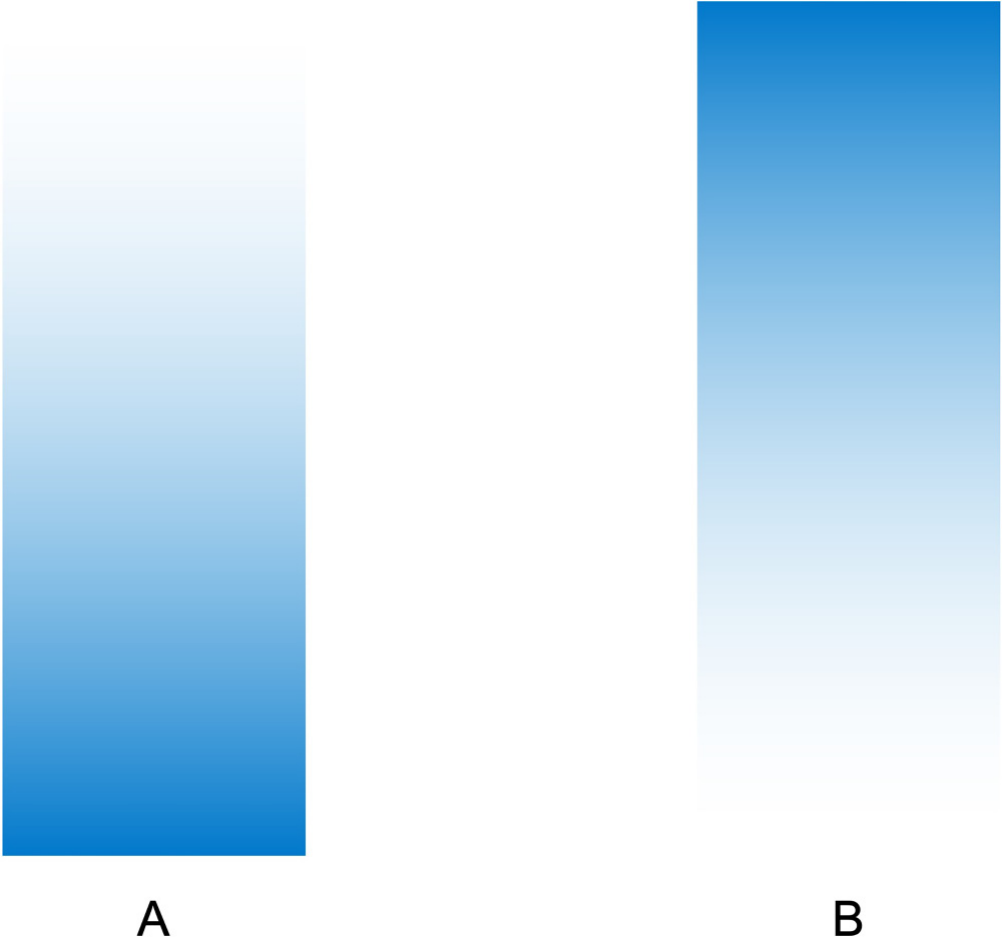
Two types of gradients. (A) Upward gradient. (B) Downward gradient.

**Figure 2.**
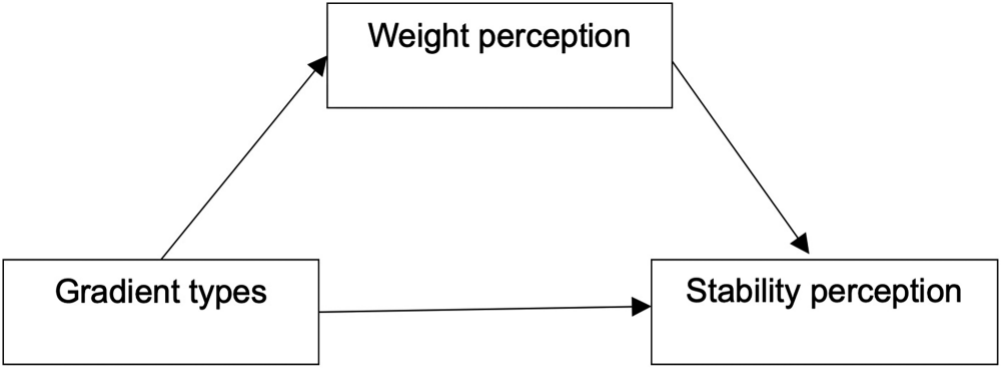
Research framework.

## Materials and Methods

### Participants

The research data were collected from Wenjuanxing (https://www.wjx.cn) in China, an online survey company specializing in providing questionnaires and data collection services. A total of 132 participants volunteered to participate in this study. The participants were 18–30 years of age or older, of whom 44 were male and 88 were female. All participants had normal visual acuity or corrected visual acuity and no color blindness or color weakness. All of them signed written informed consent. The study was approved by the academic ethics committee of the first author's university.

### Materials

**Figure 3.**
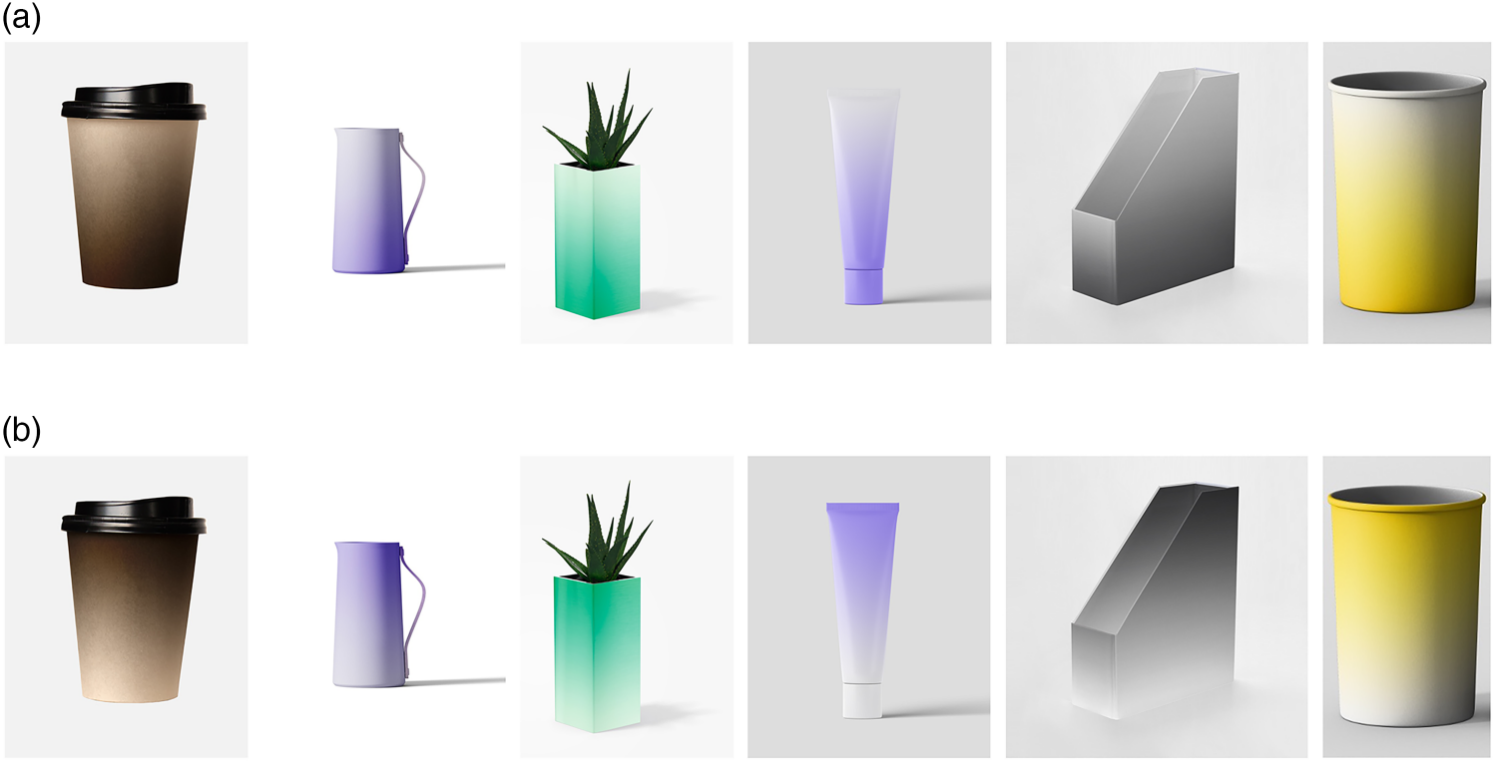
Two gradient types for six kinds of products. (A) Upward gradient. (B) Downward gradient.

In the early stage of the experiment, the participants were asked to self-report their visual acuity from the certificate of normal visual acuity or corrected visual acuity and no color blindness or color weakness. Also, the participants were asked to input the numbers seen on the plates selected from the Ishihara test. The Ishihara test is the most popular and generally considered to be the most efficient for screening red and green congenital defects (Melamud et al., 2004). The test plates are one from the “Demonstration plates” (designed to be visible by all persons, whether normal or color vision deficient. For demonstration to confirm that the participant has no other visual impairment) and two from the “Transformation plates” (individuals with color vision defect should see a different figure from individuals with normal color vision). The screening criteria for the participants are answering two of the three questions correctly to prove normal visual acuity or corrected visual acuity and no color blindness or color weakness. The stimuli consisted of six kinds of products: coffee mugs, water bottles, vases, file boxes, facial cleansing, and pen containers, all of which are common products and stability is one of the more sensitive consumer attributes when purchasing these products. Considering those aesthetics are one of the important factors influencing product consumption decisions, products with different color gradient types may produce certain aesthetic attributes (Gegenfurtner & Kiper, 2003). We used pre-experiments for material assessment. Two types of color brightness gradients (upward and downward gradient, see Figure 3) were set for the same product. The color of each product was chosen based on the most commonly used product colors in the market to simulate picking products on consumer scenarios. In total, 23 participants who had not participated in the formal experiment joined the pre-experiment, and all subjects rated the aesthetics of the six products. The evaluation was done using a 5-point Likert scale, where 1 indicates “very unattractive” and 5 indicates “very attractive.” The results revealed that the t-test for the aesthetics of the six products with two color gradient types, all with *p* > .05, were coffee mug (0.106), water bottle (0.068), vase (0.362), file box (0.315), facial cleansing (0.119), and pen container (0.170), indicating that there was no significant difference in the aesthetics of the products with the two gradient conditions, as shown in Table 1.

**Table 1.** The aesthetic evaluation of six products.

Product	Coffee mug		Water bottle		Vase		File box		Facial cleaning		Pen container	
Gradient type	A	B	A	B	A	B	A	B	A	B	A	B
*M*	3.29	3.38	3.88	3.29	3.71	3.75	3.13	2.43	3.29	3.25	3.00	2.86
*SD*	0.951	1.310	1.147	0.488	0.951	1.125	1.204	0.787	0.756	1.238	1.366	0.690
*t* test	0.106		0.068		0.362		0.315		0.119		0.170	

*Note.* (A) Upward gradient. (B) Downward gradient. *M =* mean; *SD =* standard deviation.

### Procedures

A single-factor within-subjects design was used for the experiment. The independent variable is the type of color gradient, with 2 levels, dark upward gradient (lighter on top and darker on bottom) and dark downward gradient (darker on top and lighter on bottom). The dependent variables are the stability and perception of the product. To avoid possible interactions between different gradient types of the same product in the perception assessment, we used a between-item approach, where the same participant assessed the perception of three products (e.g., coffee mug, water bottle, and vase) of one type of gradient (e.g., upward gradient) and another three products (e.g., file box, facial cleanser, and pen holder) of another type of gradient (e.g., downward gradient).

According to a given consumption scenario with particular stability requirements, the participants were asked to successively score the degree of stability of the three products with an upward gradient and the other three products with a downward gradient. The score was scored on a 7-point scale, with 1 indicating “very unstable” and 7 indicating “very stable”. After the stability assessment, the subjects were also required to measure the weight perceptions of the products. Referring to the method of Hagtvedt and Brasel (2017), this experiment required the participants to evaluate the weight of each product in kg or g within a given reasonable weight range (Hagtvedt & Brasel, 2017).

## Results

A paired samples *t-*test was used to find a significant effect of asymptotic type on perceived product stability, *t*(131) = 15.05, *p *< .001, Cohen's *d *= 2.63, indicating that perceived stability was significantly higher for the upward gradient (*M *= 5.74, *SD *= 1.37) than for the downward gradient (*M *= 2.64, *SD *= 1.56) product. Since the weight range of each type of product varies with a large gap, the number of weight perceptions was processed using a standardized treatment followed by a paired-samples *t* test, which revealed a significant effect of gradient type on product weight perception, *t*(131) = 2.46, *p *= .015, Cohen's *d *= 0.43, indicating that the weight perception of the upward gradient (*M *= 0.11, *SD *= 0.71) was significantly greater than that of the downward gradient (*M *= −0.11, *SD *= 0.73).

Using Pearson correlation analysis, a pairwise correlation was found between asymptotic type and weight perception and stability perception, as shown in Table 2.

**Table 2.** Correlation matrix for each variable.

	1	2	3	4
1.Gender	−			
2.Gradient type	−	−		
3.Weight perception	0.04	0.15 **	−	
4. Stability perception	0.22*	0.73**	0.23**	−

*Note.* *p < .05, ** *p* < .01.

**Figure 4.**
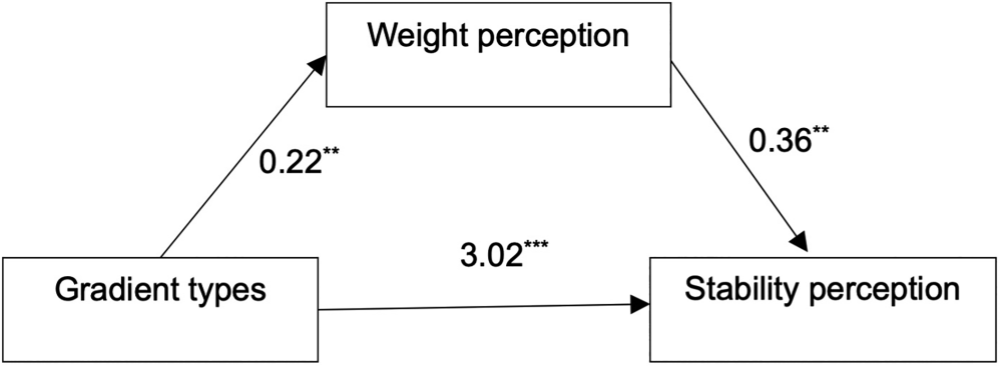
Weight percept mediated the relationship between gradient types and stability perception. *Notes.* **p < .01, ***p < .001.

Model 4 of the PROCESS macro (Hayes, 2013) was used to test the indirect effect of weight perception in the relationship between gradient types and stability perception (see Figure 4). It was found that color gradient types significantly and positively influenced stability perception (*b *= 3.02, *p *< .001) and weight perception (*b *= 0.22, *p *= .013), weight perception significantly and positively influenced stability perception (*b *= 0.36, *p *= .004), and weight perception mediated between gradient types and stability perception with a 95% CI of [0.011, 0.177] and does not contain 0; thus, the mediating effect was significant.

The results also showed the differences in stability perception between males and females, with females having a higher perception of stability compared to males, *t*(130) = 2.35, *p *= .02. Analysis of variance (ANOVA) on the effect of gender and gradient on stability assessment. It was found that the effect of gender on the estimation of stability was not significantly different, *F*(1, 130) = 0.55, *p *= .459; the effect of gender on the estimation of weight was not significantly different, either, *F*(1, 130) = 0.11, *p *= .744. However, there is a significant difference between gender and stability estimation under different gradient types, *F*(1, 130) = 5.72, *p *= .018. The simple effect test found that under type A, the gender difference is not significant, *F*(1, 130) = 1.88, *p *= .173, but under type B, the gender difference is significant, *F*(1, 130) = 6.55, *p *= .012, and the estimation of female stability (*M *= 5.95, *SD *= 1.20) were significantly higher than males (*M *= 5.32, *SD *= 1.58). This result seems to indicate that gender and gradient types jointly affect the perception of stability, with women being more sensitive to the perception of downward gradient.

## Discussion

In color psychology studies, many early studies overlooked the variables of brightness and referred only to hues (Rider, 2010). There are even fewer theoretical or empirical studies on the effect of color on the perception of stability. In the process of examining the relationship between color gradients and stability perception by manipulating the types of gradient colors, we proposed the research hypothesis that the type of monochrome brightness gradient triggers the perception of weight, which in turn affects people's perception of product stability. We first control for the effect of aesthetic differences on the experiment, and the results of the pre-experiment confirm that there is no significant difference in the assessment of product aesthetics under the different color gradient conditions of the current study. The results of the formal experiments showed that the upward gradient produced significantly higher stability perceptions than the downward gradient, and it also produced significantly higher weight perceptions than the downward gradient, and these results supported hypotheses 1 and 2. In addition, it was illustrated in the mediation test analysis that weight perception mediated the effect of color gradient on stability perception, supporting hypothesis 3. The findings, consequently, provide direction for the exploration of the little-explored relationship of color gradient type on stability perception and shed light on the underlying processes in which the effect of weight perception plays a mediating role.

Interestingly, the results also revealed differences in stability perception caused by the downward gradient between males and females, with females being more sensitive to the stability perception of the downward gradient compared to males. This may be related to the gender differences in color metaphors, also factors that influence color perception and application include gender (Singh & Srivastava, 2011; Vanston & Strother, 2017). Moreover, the significance of the mediating effect of weight perception in the process of color gradient influence on stability perception was relatively small from the experimental results, probably due to the fact that visual illusions do not always extend to the tactile realm (Hagtvedt & Brasel, 2017). When consumers evaluate an object visually, there may be a disconnect between the vision perceptual processing and the corresponding tactile information (Walker et al., 2010). Future research could investigate in depth how these issues are addressed in the minds of consumers. Additionally, extant research further indicates that emotions can enhance contrast sensitivity (Phelps et al., 2006). This demonstrates that the role of weight in the effect of stability perception may depend in part on the current emotional state of the viewer. The interaction of color perception with other sensory forms remains largely unexplored and could represent a fruitful area for further exploration.

Nevertheless, there are some limitations to this experiment. Since this experiment was conducted online, product color presentation relied on electronic methods of presentation. We cannot rule out the possibility that the color effect of the electronic presentation may vary depending on the screen of the mobile device due to differences in the environment or the hardware used by the participants. At the same time, due to the use of different shapes of products in this experiment, there might be an interaction between color and shape in the perceptual processing of evaluating object stability (Lupo, 2015). In addition to this, however, our experiments suffer from the general limitations of color research—it is difficult to ensure that the experimental stimuli reflect the exact attributes specified (Hagtvedt & Brasel, 2017), since the effect of color on people's mental perception is usually unconsciously perceived (Löffler, 2017). As this experiment was in the form of an online questionnaire, in-depth interviews were not conducted with each subject regarding the motivation or reason for the choice to further confirm that the mechanism behind the effect was true as shown in the experimental results. Future research could also focus on the extent to which the color effect relies on automatic or conscious processes.

Very little research has so far focused on combined color effects (Deng et al., 2010). The study focuses on the brightness and there is no combining such as hues to prove if this effect works equally well for all hues. Future research could continue to explore the relationship between different hue gradients (e.g., red → cyan, yellow→blue) and stability. Furthermore, the inconsistent use of color terminology in the current literature may complicate the interpretation of study results. The terms brightness and value, lightness and illuminance are used interchangeably, but sometimes have different meanings (Labrecque et al., 2013; Stone et al., 2006; Walker et al., 2010). As these situations arise, it is difficult and challenging to interpret research results using a series of terms and concepts that are difficult to clearly define. As a researcher, we need to be clear about the concepts referred to by the terms involved in our research discourse so that they can be used for comparison with future research.

### Conclusions

In this study, the experimental method was used to investigate the effect of color gradient type (dark color fading upward versus downward) on weight perception and stability perception, and it was found that dark color fading upward (the color gradient dark color from bottom to light color on top) brought stronger stability perception and weight perception than dark color fading upward (the color gradient dark color from top to light color in the bottom). Moreover, weight perception mediates the effect of both gradient types on product stability perception. These findings are of broad practical relevance. As a powerful medium for people to observe the world (Rider, 2010), color has a profound effect on human perception, preference, and psychology. For psychologists, the subtle and powerful influence of color is a fascinating field of study realm (Elliot & Maier, 2007). As a marketing tool, color has been widely used to attract consumers’ attention and influence their perceptions of products. Mohebbi (2014) illustrates the key role that color plays in packaging design to influence consumers’ purchasing decisions, emphasizing the importance of color in marketing (Mohebbi, 2014). Color is a tool for designers to communicate with consumers, whether the dialogue is conscious or unconscious, using color in the process of marketing practice is an effective method to implement it (Gabbas et al., 2021; Rider, 2010). For example, if we want to influence consumer perceptions of product weight and stability, gradient color is a relatively low-cost way to do so (Burke, 2000). Considering the field of application, with the growing popularity of online shopping, and gradient color as a product eye-catching visual performance design method. How to correctly use gradient color to enhance consumer perception of the physical nature of the product seems very important, especially in the online shopping, as the visual impression may be the only sensory measurement available to consumers in the digital environment (Hagtvedt & Brasel, 2017).
